# Fear and death anxiety in the shadow of COVID-19 among the Lebanese population: A cross-sectional study

**DOI:** 10.1371/journal.pone.0270567

**Published:** 2022-07-27

**Authors:** Zeinab Chalhoub, Hiba Koubeissy, Youssef Fares, Linda Abou-Abbas

**Affiliations:** Neuroscience Research Center, Faculty of Medical Sciences, Lebanese University, Beirut, Lebanon; Iranian Institute for Health Sciences Research, ISLAMIC REPUBLIC OF IRAN

## Abstract

**Background:**

The COVID-19 pandemic was one of the most devastating health crises the world has seen. One of its mental health consequences includes fear of being infected, which could lead to anxiety. This study aimed to assess the fear related to the COVID-19 pandemic and its associated factors among the adult population in Lebanon.

**Methods:**

A cross-sectional study was conducted in Lebanon between February 26^th^ and April 29^th^, 2021. Data was collected through an online survey among adults aged 18 years and older. Information on sociodemographic and clinical characteristics of the participants, fear and death anxiety related to the COVID-19 pandemic, depression, and anxiety were collected. Multivariable linear regression analyses were carried out to identify the predictors of fear related to the COVID-19 pandemic.

**Results:**

A total of 1840 participants were included in the analysis of which 62.9% were females and 62.2% were single. The age of the participants ranged from 18 to 70 years with a mean of 26.6 ±8.8 years. Of the total participants, 41.9% felt uncomfortable thinking about the novel Coronavirus and 35.4% of candidates became nervous/anxious when watching the news about COVID-19 on social media. About one-third of the participants (33.7%) were afraid of COVID-19 and 23.8% were afraid of losing their life because of the disease. Concerning somatic symptoms of fear, 7.9% reported increased heart races or palpitations whenever they thought about getting infected with COVID-19, 3.7% complained about sleep disturbances while 2.5% developed tremors or sweating in their hands when they thought about Coronavirus. In addition, Death anxiety related to the COVID-19 pandemic was one of the most fear-related factors (B = 0.191, 95% CI (0.172 to 0.211), P-value < 0.0001).

**Conclusion:**

This study provides insights on the impact of COVID-19 on individuals at the level of fear in Lebanon. Death anxiety was identified as the most significant predictor of fear related to the COVID-19 pandemic. Considering the negative psychological effects of fear, it is necessary to educate the adults on how to deal with death anxiety and implement psychological interventions and counseling programs to relieve fear and improve the mental health of Lebanese adults.

## Introduction

Coronavirus disease 2019 (COVID-19), which was first identified in Wuhan China in December 2019, had rapidly spread all over the world and swiftly became a global health threat [1]. At the time of writing, more than 207 million confirmed cases related to COVID-19 had been registered worldwide with more than 4 million deaths [2]. COVID-19 is accompanied by a lot of symptoms ranging from mild to severe with worse outcomes affecting people with comorbidities. In addition to its physical impacts, COVID-19 can have serious effects on people’s mental health of the infected patients as well as the health care workers and the general population [3–5]. A wide range of psychological outcomes has been observed during this pandemic such as emotional distress, depression, stress, mood swings, irritability, insomnia, attention deficit hyperactivity disorder, post-traumatic stress, and anger [4, 6].

Health crises such as the COVID-19 pandemic are initiated by fear of being infected which in turn can reinforce the anxious state [7]. Fear is an essential adaptive response while dealing with a potential threat. However, it can be maladaptive in case of incompatibility with the actual threat. Excessive fear may alarm humans and society so that they will be stick to the norms of safety, but it may paradoxically also reinforce the negative fear that may lead to obsessions such as contamination concerns or compulsions that are focused on maintaining safety [8, 9]. In addition to enhanced fear, death anxiety could be stimulated due to diseases with high mortality rates [10, 11]. Since COVID-19 has killed many people all around the world, individuals’ levels of death anxiety due to COVID-19 are likely to be promoted often resulting in increasing the levels of fear during the pandemic.

Death anxiety, also known as thanatophobia, is anxiety produced by thoughts of one’s own death (fear of death). Its negative impacts have been shown in various areas of people’s lives such as pessimism, despair, poor understanding of social support, and insufficient life perception [12]. Such stressful conditions make the person unable to process properly and experience severe psychological suffering [13]. Social support may help to reduce death anxiety by increasing people’s sense of belonging to the social context and their belief in their ability to cope with challenges [14, 15]. In the context of death anxiety, different cultural worldviews exist and have a noticeable influence on the degree to which the people fear death. Thus, clarifying the impact of death anxiety on fear in different population is important and can assist health decision makers in dealing with this issue more effectively. In Lebanon, confirmed COVID-19 cases continue to rise; now exceeding 500,000 cases. In addition, more than 7600 deaths were recorded by 16 August 2021 [15]. Combined with an economic collapse, the pandemic has overburdened the public health system and has provoked catastrophic consequences on people’s livelihoods and mental health [16]. As the pandemic continues to run its course, it is recommended to continue monitoring the mental health consequences of this pandemic over time especially among elder people and vulnerable persons [17]. Thus, the Ministry of Public Health (MOPH) through the National Mental Health Program launched an action plan to address mental health and psychosocial aspects of the COVID-19 Outbreak as part of the national response to the pandemic. However, limited data examining the impact of COVID-19 on the mental health status of the Lebanese population were published [16, 18]. Available studies indicated higher levels of stress, depression, and anxiety, as well as lower obsessive-compulsive traits among the Lebanese general population [18]. As fear may be an essential construct in explaining these negative psychological consequences of the COVID-19 pandemic, it is important to pinpoint what people are exactly afraid of and identify relevant predictors. So far, fear related to the COVID-19 pandemic was not assessed in the Lebanese general population. Thus, we sought to assess the fear related to the COVID-19 pandemic among the adult population in Lebanon. Another goal of our study was to investigate possible predictors of increased fear of the COVID-19 pandemic. Several possible predictors of COVID-19 fear can be derived from the literature. One predictor of interest in our study is death anxiety. Given that the pandemic is not over yet and the number of deaths is still increasing, we expected that death anxiety is a significant predictor for increased fear of the coronavirus. The results of this study may provide health policymakers with relevant information about modifiable factors to optimize the effectiveness of mental health support programs.

## Methods

### Study design and population

A cross-sectional study was conducted during the rapid rise phase of the COVID-19 epidemic in Lebanon between February 26^th^ and April 29^th^, 2021. All Lebanese adults aged 18 years and above, able to read and understand the Arabic language, and who agreed to participate in the study were eligible to participate in the study. Due to this pandemic, which obliged the Lebanese government to ratify the curfew law and to reduce direct interaction between people, it has become customary to invite participants using electronic questionnaire. Thus, a data collection tool built in Google forms was distributed to the participants using a snowball technique via social media applications “WhatsApp, Facebook and Telegram”.

### Ethical consideration

The study protocol was reviewed and approved by the scientific committee of the Neuroscience Research Center (NRC) at the Faculty of Medical Sciences, Lebanese University. At the beginning of the Google form, Participants were informed about the objectives of the study and they were asked to sign a participation consent form. They were also informed that they have the right to refuse to participate at any time and this will involve neither a penalty nor a risk. Anonymity and confidentiality of information were respected.

### Sample size calculation

The sample size was calculated using the online sample size “Raosoft^®^” calculator. Based on an estimated Lebanese population of 4.6 million, a 5% margin error, and a 95% confidence level, the minimum sample size would be 384.

### Instrumentation

Data was collected via an online questionnaire developed by Google forms and made available in Arabic language.

The questionnaire was divided into several parts:

1. **Baseline characteristics** such as age, gender, education level, marital status, work status, family income per month, health status, family history of chronic disease, previous infection by COVID-19, psychotherapist consultation due to the outbreak and spread of the COVID-19, adherence to home quarantine and the required recommendations during the lockdown period and their willingness to receive Covid-19 vaccine.
2. **Fear of COVID-19 infection:** The Arabic version of the Fear of COVID-19 Scale (FCV-19S) was used to evaluate the Fear of COVID-19 infection. The FCV-19S is a tool that has been developed by Ahorsu et al to measure one’s fear of COVID-19 [19]. It consists of 7 items and the response to each item can be graded on a Likert scale, ranging from 1 (strongly disagree) to 5 (strongly agree). A total score is calculated by summing all item scores with a possible total score ranging between 7 and 35. Higher scores indicate greater levels of fear of COVID-19. The Arabic version of FCV-19S showed a unidimensional structure, good internal consistency, good concurrent validity, and acceptable construct validity [20].
3. **Death anxiety:** The Arabic scale of death anxiety (ASDA) was used to evaluate the Death anxiety [21]. It consists of 20 items and the response to each item can be graded on a Likert scale, ranging from 1 (no) to 5 (very much). A total score is calculated by summing all item scores with a possible total score ranging between 20 and 100. Higher scores indicate greater levels of death anxiety. The Arabic Scale of Death Anxiety (ASDA) was constructed and validated in a sample of undergraduates (17–33 yrs) in 3 Arab countries, Egypt (n = 418), Kuwait (n = 509), and Syria (n = 709). Alpha reliabilities ranged from 0.88 to 0.93, and the 1-week test-retest reliability was 0.90 (Egyptians only), denoting high internal consistency and stability [21].
4. **Depression** was assessed using “the Patient Health Questionnaire (PHQ9)” which is a 9-item self-report reliable and valid measure of depression severity [22]. The response to each item can be graded on a Likert scale from 0 “absence of symptom” to 3 “presence of symptom nearly every day”. The total score was computed by adding the scores of the 9 items with higher scores indicating a higher level of depression. PHQ9 is a valid and reliable tool among the Lebanese population [23].

**Anxiety** was assessed using “the Generalized anxiety disorder (GAD-7)” which consists of 7 items rated on a four-point Likert scale ranging from 0 “absence of symptom” to 3 “presence of symptom nearly every day. A total score is computed by adding the scores of the 7 items with higher scores indicating a higher level of anxiety. GAD-7 has proven to be a useful measure with strong psychometric properties like criterion validity, construct validity, reliability, test-retest reliability, factorial and procedural validity [24]. GAD-7 has also been validated among the Lebanese population and it was found to be a valid and reliable tool [23].

### Pilot study

The questionnaire was pretested in a sample of 32 participants, divided into two groups, to check its clarity and readability. The first group of participants reported some problems concerning the clarity and the comprehensibility of some scales used in the questionnaire; also, they reported questionnaire long duration. Thus, the questionnaire has undergone some modification and some items were eliminated and a second version was made and shared with the second group, who did not report any problem concerning the clarity and the comprehensibility and the time required to fill out the questionnaire. All the questions were completed within approximately 10 minutes.

### Statistical analysis

Data entry and analysis were done using SPSS software, version 22. Descriptive statistics were reported using means and standard deviations (SD) for variables with adequate normal distribution, and frequency (n) with percentages (%) for categorical variables. Since no published data were available on the validity of the FCV-19S in the Lebanese context at the time of writing, we evaluated the FCV-19S factor structure using a Principle Components Analysis (PCA). Sampling adequacy was assessed by the Kaiser–Meyer–Olkin (KMO) measure and Bartlett’s test of sphericity. The number of factors retained in the scale was determined based on Eigenvalues greater than 1, and visual inspection of the scree plot. Additionally, we conducted the parallel analysis using an existing syntax written in SPSS [25]. It was based on random data generation, which is parallel to the actual dataset, and accordingly, the number of factors was determined. In addition, Cronbach’s alpha was calculated to assess the internal consistency of the FCV-19S. A coefficient of above 0.7 indicated a good internal consistency. Then, predictors of FCV-19S were investigated using Spearman’s correlation coefficients for the continuous predictors, Student T-test, Manwhitney, one-way ANOVA, and Kruskal Wallis for the categorical predictors. Following bivariate analyses, ordinary least square linear regression was conducted including all significant predictors from the bivariate analyses to investigate factors significantly predicting fear of COVID-19. Linearity of the relationship, the normality of distribution of residuals, and the non-colinearity of retained variables were insured before the model was accepted. Unstandardized regression coefficients (B) with their 95% confidence intervals were computed to present the results of multivariate analysis. A p-value of less than 0.05 was considered statistically significant.

## Results

### Baseline characteristics of the studied population

Table 1 displays the baseline characteristics of the study participants. The proportion of valid questionnaires was 91.4%. A total of 1840 participants were included in the analysis of which 62.9% were females and 62.2% were single. The age of the participants ranged from 18 to 70 years with a mean of 26.6 ±8.8 years. The majority of respondents (85.1%) had more than 12 years of education and 55.5% were unemployed. Additionally, 697(37.9%) of the total sample had a monthly family income ranged from 1–2 million LL. Concerning the clinical characteristics of the sample, the majority of the participants (93.1%) had a good to excellent health status and approximately half of them (48.6%) had a family history of chronic diseases. At the time of the survey, 77% of the participant weren’t infected by COVID-19 and 92.6% was abiding to the home quarantine and the required recommendations during the lockdown period. From the total sample, 3.6% of participants sought or consulted a psychotherapist as a result of the COVID-19 pandemic, and 66.3% were willing to receive the COVID-19 vaccine upon availability.

**Table 1:** Baseline characteristics of the study participants (N = 1840).

Variable	All
**Age (Mean ±SD) (years)**	26.6±8.8
**Gender n (%)**	
Female	1157 (62.9)
Male	683 (37.1)
**Marital status n (%)**	
Single	1144 (62.2)
Others	696 (37.8)
**Educational level n (%)**	
≤12 years	275 (14.9)
>12 years	1565 (85.1)
**Work status n (%)**	
No	1021 (55.5)
Yes	819 (44.5)
**Family income per month n (%)**	
≤ 1 million LL	300 (16.3)
1–2 million LL	697 (37.9)
2–4 million LL	477 (25.9)
˃4 million LL	366 (19.9)
**Health status n (%)**	
Bad	128 (7.0)
Good to Excellent	1712 (93.0)
**Family history of chronic disease n (%)**	
No	946 (51.4)
Yes	894 (48.6)
**Previous infection with COVID-19 n (%)**	
No	1416 (77.0)
Yes	424 (23.0)
**Psychotherapist Consultation due to COVID-19 n (%)**	
No	1773 (96.4)
Yes	67 (3.6)
**Adherence to safety measures during the lockdown period n (%)**	
No	137 (7.4)
Yes	1703 (92.6)
**Willingness to receive the COVID-19 vaccine n (%)**	
No	620 (33.7)
Yes	1220 (66.3)

n: frequency, %: percentage, SD: standard deviation.

### Validity and reliability of the scales

The exploratory factor analysis of the FCV-S19 scale in our sample showed a KMO measure of 0.846 indicating adequate sampling adequacy, a highly significant Bartlett’s test of sphericity (χ2 = 5231.023, df = 21, p-value <0.0001). The scree plot of the Eigenvalues revealed a two-factor structure of the FCV-S19 scale which explained 68.4% of the FCV-S19 variance. Additionally, the results of the parallel analyses showed that the eigenvalue of the actual dataset only exceeded the eigenvalue of the simulated dataset for two factors. Based on these results, we decided on a two-factor structure for the FCV-S19: the first factor was related to worry of COVID-19 (item 1, 2, 4 & 5) and accounted for 53.0% of the scale’s total variance, the second factor was associated with somatic symptoms of fear and accounted for 15.4% of the scale’s total variance (items 3, 6 & 7).

The validation of the ASDA questionnaire in our sample revealed good psychometric properties of the scale. High internal consistency with a Cronbach’s alpha = 0.955 was found. In addition, exploratory analysis extracted 3 factors: Factor 1 expressed the fear of dead people and tombs, the second factor was associated with fear of postmortem events and death concern, and the third factor concerned fear of lethal disease.

The internal consistency coefficient of the PHQ-9 and GAD-7 scale were also calculated. Results revealed was good Cronbach’s alpha of 0.883 and 0.936 for PHQ-9 and GAD-7 respectively.

### Fear of COVID-19 among the study participants

Of the total participants, 41.9% felt uncomfortable thinking about the novel Coronavirus and 35.4% of candidates became nervous/anxious when watching the news about COVID-19 on social media. About one-third of the participants (33.7%) were afraid of COVID-19 and 23.8% were afraid of losing their life because of the disease. Of the total participants, 7.9% reported increased heart races or palpitations whenever they thought about getting infected with Covid-19, 3.7% complained about sleep disturbances while 2.5% developed tremors or sweating in their hands when they thought about the Coronavirus. The mean score of the FCV-19S total score was 15.6 ± 5.9 (Table 2).

**Table 2:** Fear of COVID-19 among the study participants.

FCV-19S item	Strongly disagree n(%)	Disagree n(%)	Neutral n(%)	Agree n (%)	Strongly agree n(%)
I am most afraid of Corona	325(17.7)	349(19.0)	546(29.7)	425(23.1)	195(10.6)
It makes me uncomfortable to think about Corona	35(18.8)	361(19.6)	364(19.8)	458(24.9)	312(17.0)
My hands become clammy when I think about Corona	1459(79.3)	252(13.7)	82(4.5)	28(1.5)	19(1.0)
I am afraid of losing my life because of Corona	644(35.0)	359(19.5)	342(18.6)	260(14.1)	234(12.8)
When I watch news and stories about Corona on social media, I become nervous or anxious	438(23.8)	395(21.5)	355(19.3)	401(21.8)	25(13.6)
I cannot sleep because I’m worrying about getting Corona	1405(76.4)	244(13.3)	123(6.7)	42(2.3)	26(1.4)
My heart races or palpitates when I think about getting Corona	1208(65.7)	296(16.1)	19(10.4)	77(4.2)	68(3.7)
FCV-19S Total score Mean ±SD	15.60 ±5.9				

FCV-19S Fear of COVID-19 scale, n frequency, % percentage, SD Standard Deviation.

### Factors associated with fear of COVID-19 infection among the Lebanese population

Bivariate analyses were conducted to explore the factors associated with FCV-19S among the study participants. Results showed that gender, marital status, work status, health status, Family history of chronic disease, previous infection with COVID-19, PHQ-9, GAD-7, and ASDA were significantly associated with FCV-19S (Table 3). Multiple regression analysis was then conducted to determine the predictors of the fear of COVID-19 infection. The significant predictors were ASDA (B = 0.191, 95% CI (0.172 to 0.211), P-value < 0.0001), GAD-7 (B = 0.195, 95% CI (0.155 to 0.236), P-value < 0.0001), marital status (B = 1.604, 95% CI (1.152 to 2.055), P-value < 0.0001), previous infection with COVID-19 (B = -0.742, 95% CI (-1.259 to -0.224), P-value = 0.005), and family history of chronic disease (B = 0.463, 95% CI (0.022 to 0.904), P-value = 0.039) in other words, persons with higher scores of death anxiety and anxiety (GAD-7) had a higher level of fear of COVID-19. Married or divorced participants and those having a family history of chronic diseases had also a higher level of fear of COVID-19 infection compared to their counterparts. However, people previously infected by COVID-19 were less likely to suffer from fear of COVID-19. In addition, fear of COVID-19 infection can affect positively the decision for taking the COVID-19 vaccine (B = 1.222, 95% CI (0.756 to 1.688), p-value<0.0001), increase the level of psychotherapist consultation (B = 2.966, 95% CI (1.795 to 4.138), p-value<0.0001) and in a positive way increase the level of the adherence to safety measures during the lockdown period (B = 2.198, 95% CI (1.358 to 3.038), p-value<0.0001). This model explained 36% of the variance in the FCV-19S. It is also worth noting that the death anxiety scale assessed by ASDA was the best predictor of the fear of COVID-19 (Standardized B = 0.410) followed by the GAD-7 (Standardized B = 0.203) (Table 4).

**Table 3:** Bivariate analyses investigating the possible predictors of the FCV-19S among the study participants (N = 1840).

Categorical Variables	Mean (SD)	P-value
**Gender**		<0.0001
Female	16.58±6	
Male	13.85±5.4	
**Marital status**		0.002
Single	15.23±5.9	
Others	16.11±6	
**Educational level**		0.618
≤12 years	15.74±6.5	
>12 years	15.53±5.8	
**Work status**		<0.0001
No	16.06±6	
Yes	14.94±5.8	
**Family income per month**		0.116
≤ 1 million LL	16±6.7	
1–2 million LL	15.8±6	
2–4 million LL	15.36±5.6	
˃4 million LL	15.05±5.5	
**Health status**		0.001
Bad	17.59±7.3	
Good to Excellent	15.41±5.8	
**Family history of chronic disease**		<0.0001
No	14.98±5.7	
Yes	16.18±6	
**Previous infection with COVID-19**		<0.0001
No	15.84±5.9	
Yes	14.66 ±6	
**Psychotherapist Consultation due to COVID-19**		<0.0001
No	15.39±5.8	
Yes	20.13±7.3	
**Adherence to safety measures during the lockdown period**		<0.0001
No	12.02±5	
Yes	15.85±6	
**Willingness to receive the COVID-19 vaccine**		
No	14.4±5.9	<0.0001
Yes	16.16 ±5.9	
**Continuous Variables**	**r**	**P-value**
**Age**	0.03	0.193
**PHQ-9 score**	0.32	<0.0001
**GAD-7 score**	0.4	<0.0001
**ASDA score**	0.54	<0.0001

SD: standard deviation, r correlation coefficient, ASDA: Arabic scale of death anxiety, GAD-7: The Generalized anxiety disorder, PHQ-9: The Patient Health Questionnaire (PHQ9).

**Table 4:** Factors associated with the COVID-19 infection fear.

FCV-19S					
	Unstandardized Coefficients	Standardized Coefficient	P-value	95% Confidence Interval for B	
Model	B	Beta		Lower Bound	Upper Bound
ASDA	0.191	0.410	<0.0001	0.172	0.211
GAD-7	0.195	0.203	<0.0001	0.155	0.236
Marital status	1.604	0.131	<0.0001	1.152	2.055
Willingness to receive the COVID-19 vaccine	1.222	0.097	<0.0001	0.756	1.688
Adherence to safety measures during the lockdown period	2.198	0.097	<0.0001	1.358	3.038
Psychotherapist Consultation due to COVID-19	2.966	0.094	<0.0001	1.795	4.138
Previous infection with COVID-19	-0.742	-0.053	0.005	-1.259	-0.224
Family history of chronic disease	0.463	0.039	0.039	0.022	0.904

FCV-19S: fear of COVID-19 scale, ASDA: Arabic scale of death anxiety, GAD-7: The Generalized anxiety disorder

## Discussion

The present cross-sectional study was carried out among Lebanese individuals to investigate the fear of COVID-19 and its association with death anxiety during the rapid rise period of the coronavirus pandemic in Lebanon. Results of the present study showed variability in participants’ agreement on the seven items of the COVID-19 fear scale with a high prevalence for the items that expressed the fear and worry about the COVID-19 pandemic and low prevalence for the items related to the somatic symptoms of fear. This can be explained by the fact that COVID-19 was a global pandemic that caused fear all over the world and this has resulted in people being able to express their feelings of fear without embarrassment.

Our results replicate the findings from other studies [26–28]. Particularly, a sizable proportion of adults reported a prevalence of agreement ranging between 23.8%-41.9% on the FCV-19S items related to worry of COVID-19. We found out that 41.9% felt uncomfortable thinking about the novel Coronavirus, 33.7% were afraid of COVID-19 and 23.8% were afraid of losing their life because of the disease. The rapid community spread of the disease, the fear of self-infection or infection of family members or loved ones, the rising number of deaths, the mistrust of the health care system, and the misleading information concerning life-threatening aspects of COVID-19 may have all contributed significantly to the increased level of worries and fear of COVID-19 among Lebanese adult population which can result in specific phobias, and psychological distress [24]. Furthermore, 35.4% of the participants felt nervous or anxious when watching or hearing news of COVID 19 in any form of social media. Although social media provides an easy means for getting information, it can expose their followers to a massive amount of fake and unreliable news that may explain their high level of anxiety and fear. Studies conducted during previous disease outbreaks such as avian influenza (H5N1) revealed that exposure to mass media is highly associated with worrying about the virus [29]. As such, we expect that for the coronavirus pandemic, more media coverage of threat information related to the pandemic would increase the level of fear. Based on our findings, it would be recommended for health policymakers to adopt new strategies for community and individuals level about the pandemic by running campaigns focusing on delivering accurate and evidence-based information to minimize the effect of fake news and raising awareness about the factors affecting the risk of COVID-19, in addition to the training provided for its prevention and effective coping strategies.

The present study also identified the factors associated with COVID-19 related fear among the Lebanese adult population. Results found that death anxiety related to the COVID-19 pandemic was one of the most fear-related factors. Although, few studies have investigated the association between fear and death anxiety, a recent study that evaluated the psychometric properties of the fear scale (FCV-19S) by Ahorsu et al. reported that the fourth item ‘I am afraid of losing my life because of coronavirus-19’ had the highest factor loading suggesting that worries about the lethality of the COVID-19 are highly predictive of fears of the virus [19].

In our study, general anxiety assessed by GAD-7 was also found to be a significant predictor of fear of COVID-19. This comes in line with the results of the study conducted in Iran [19]. Fear and anxiety caused by the possible disease can impose a high and destructive psychological burden, leading to mental disorders, weakening the immune system, and reducing the body’s ability to fight disease in people [30].

Married and divorced participants also showed a higher level of Fear of COVID-19 infection compared to single persons. This can be considered an expected result as married and divorced people could care more about people they loved (e.g. husband and children). But on the other side, this result was not consistent with the ones reported in a recent study conducted in Saudi Arabia which revealed that divorced and unmarried persons present a higher level of fear of infection [27]. In addition, persons who were willing to take the vaccine were adherent to safety measures during the lockdown period and those who seek psychotherapist consultation due to COVID-19 had a higher level of fear of COVID-19 infection compared to their counterparts. Interestingly, persons that weren’t previously infected by COVID-19 were more likely to present a high level of fear of COVID-19 infection than their counterparts. Finally, the presence of family member with the chronic disease increases the level of fear of COVID-19 infection, this is an expected result since a patient with chronic disease are more at risk to suffer from drastic outcomes resulting from the infection by COVID-19, which increase the level of fear of infection [31].

To the best of our knowledge, this is the first study exploring the fear of COVID-19 and its association with death anxiety among the adult population in Lebanon. This survey was the largest one on COVID-19 conducted in Lebanon during the 2021 rapid rise period of the pandemic. Strengths of the present study include the high response rate and the used of well-validated tools to assess fear and death anxiety related to the COVID-19 pandemic; however, our study has some limitations: Selection bias is possible due to the snowball technique that was used to collect data which limits the generalizability of the findings. Future research should use purposive or random sampling methods. The collected data was based on self-reported information which makes it prone to social desirability and recall biases. Different data gathering methods (e.g., observations and interviews) can be used in future study to overcome this constraint. Furthermore, the cross-sectional design of this survey does not allow us to draw to determine the mechanism or the cause-effect relationships.

## Conclusion

This study provides insights on the impact of COVID-19 on individuals at the level of fear in Lebanon. Death anxiety was identified as the most significant predictor of fear related to the COVID-19 pandemic. Considering the negative psychological effects of fear, it is necessary to educate the adults on how to deal with death anxiety and implement psychological interventions and counseling programs to relieve fear and improve the mental health of Lebanese adults. Future work should focus on exploring strategies to minimize the fear and death anxiety associated with the COVID-19 pandemic.

## Supporting information

S1 File Online survey instrument administered. (PDF)

S2 File Study data. All patient results and demographics information.
(XLSX)
