# Supplementary material for: Fear and death anxiety in the shadow of COVID-19 among the Lebanese population: A cross-sectional study
Source: PLoS One. 2022 Jul 27;17(7):e0270567. doi: 10.1371/journal.pone.0270567 (PMC9328531; doi:10.1371/journal.pone.0270567)
Supplement: S1 File — (PDF) [file pone.0270567.s001.pdf]

Fear and Death Anxiety in the Shadow of COVID-19 among the Lebanese Population: A Cross-sectional Study

1- Sociodemographic and clinical characteristics

**1- Age (years)**	
**2- Gender**	- Female<br>- Male
**3- Marital status**	- Single<br>- Others
4- **Educational level**	- ≤12 years<br>- >12 years
5- **Work status?**	- No<br>- Yes
6- **Family income per month**	- ≤ 1 million LL<br>- 1-2 million LL<br>- 2-4 million LL<br>- >4 million LL
7- **Health status**	- Bad<br>- Good to Excellent
8- **Family history of chronic disease**	- No<br>- Yes
**9- Previous infection with COVID-19**	- No<br>- Yes
10- **Psychotherapist Consultation due to COVID-19**	- No<br>- Yes
11- **Adherence to safety measures during the lockdown period**	- No<br>- Yes
12- **Willingness to receive the COVID-19 vaccine**	- No<br>- Yes

2- Fear of COVID-19 Scale

Please respond to each item by ticking (√) one of the five (5) responses that reflects how you feel, think or act toward COVID-19.

Fear of COVID-19 Items	Strongly Disagree	Disagree	Neutral	Agree	Strongly

					Agree
1- I am most afraid of Corona	1	2	3	4	5
2- It makes me uncomfortable to think about Corona	1	2	3	4	5
3 - My hands become clammy when I think about Corona	1	2	3	4	5
4- I am afraid of losing my life because of Corona	1	2	3	4	5
5 - When I watch news and stories about Corona on social media, I become nervous or anxious.	1	2	3	4	5
6 - I cannot sleep because I'm worrying about getting Corona.	1	2	3	4	5
7 - My heart races or palpitates when I think about getting Corona	1	2	3	4	5

3- Arabic scale of death anxiety (ASDA)

Instructions: Read the following statements, and then decide to what extent each one describes your feelings, behavior, and opinions. Show how far it does, or does not, apply to you in general by circling the appropriate number after each statement.

	No	A little	A fair amount	Much	Very Much
1- I fear death whenever I become ill.	1	2	3	4	5
2- I fear looking at the dead.	1	2	3	4	5
3- I fear visiting graves.	1	2	3	4	5
4- The possibility of having a surgical operation terrifies me.	1	2	3	4	5
5- I am afraid of suffering a heart attack.	1	2	3	4	5
6- I worry that death may deprive me of someone dear to me.	1	2	3	4	5
7- I am apprehensive of unknown things after death.	1	2	3	4	5
8- I am afraid of looking at a corpse.	1	2	3	4	5

9- I fear the torture of the grave.	1	2	3	4	5
10- I fear getting a serious disease.	1	2	3	4	5
11- Witnessing the burial procedure terrifies me.	1	2	3	4	5
12- I dread walking in graveyards.	1	2	3	4	5
13- I am preoccupied with thinking about what will happen after death.	1	2	3	4	5
14- I am afraid of sleeping and not waking up again .	1	2	3	4	5
15- The pain accompanying death terrifies me .	1	2	3	4	5
16- I get upset by witnessing a funeral.	1	2	3	4	5
17- The sight of a dying person frightens me	1	2	3	4	5
18- Talking about death upsets me.	1	2	3	4	5
19- I am afraid of getting cancer	1	2	3	4	5
20- I fear death	1	2	3	4	5

4- PATIENT HEALTH QUESTIONNAIRE-9 (PHQ-9)

Over the last 2 weeks, how often have you been bothered by any of the following problems?				
	Not at all	Several days	More than half the days	Nearly every day
1. Little interest or pleasure in doing things	0	1	2	3
2. Feeling down, depressed, or hopeless	0	1	2	3
3. Trouble falling or staying asleep, or sleeping too much	0	1	2	3
4. Feeling tired or having little energy	0	1	2	3
5. Poor appetite or overeating	0	1	2	3

6. Feeling bad about yourself — or that you are a failure or have let yourself or your family down	0	1	2	3
7. Trouble concentrating on things, such as reading the newspaper or watching television	0	1	2	3
8. Moving or speaking so slowly that other people could have noticed? Or the opposite — being so fidgety or restless that you have been moving around a lot more than usual	0	1	2	3
9. Thoughts that you would be better off dead or of hurting yourself in some way	0	1	2	3

5- the Generalized anxiety disorder (GAD-7)

Over the last 2 weeks, how often have you been bothered by any of the following problems?				
	Not at all	Several days	More than half the days	Nearly every day
1. Feeling nervous, anxious or on edge	0	1	2	3
2. Not being able to stop or control worrying	0	1	2	3
3. Worrying too much about different things	0	1	2	3
4. Trouble relaxing	0	1	2	3
5. Being so restless that it is hard to sit still	0	1	2	3
6. Becoming easily annoyed or irritable	0	1	2	3
7. Feeling afraid as if something awful might happen	0	1	2	3

الخوف من الإصابة بكوفيد-19 و القلق من الموت في ظل جائحة كورونا لدى الشعب اللبناني

1- الخصائص الديمغرافية والسريرية

1- العمر	....
2- الجنس	- ذكر ☐<br>- انثى ☐
3- الوضع العائلي	- أعزب ☐<br>- غيرها ☐
4- المستوى العلمي	- أقل من 12 سنة دراسية ☐<br>- أكثر من 12 سنة دراسية ☐
5- هل تعمل ؟	- أعمل ☐<br>- لا أعمل ☐
6- الراتب الشهري لاسرتك	- أقل من مليون ليرة لبنانية ☐<br>- 1-2 مليون ليرة لبنانية ☐<br>- 2-4 مليون ليرة لبنانية ☐<br>- أكثر من 4 مليون ليرة لبنانية ☐
7- الحالة الصحية	- سيئة ☐<br>- جيدة الى ممتازة☐
8- هل يعاني أحد أفراد أسرتك من مرض مزمن	- نعم ☐<br>- كلا ☐
9- هل اصبت بفيروس كوفيد 19(كورونا) ؟	- نعم ☐<br>- كلا ☐
10- هل استعنت او قمت باستشارة معالج نفسي نتيجة تفشي و انتشار فيروس كورنا ؟	- نعم ☐<br>- كلا ☐
11- هل التزمت بالحجر المنزلي و التوصيات المطلوبة خلال فترة الإقفال العام؟	- نعم ☐<br>- كلا ☐
12- هل ترغب في تلقي لقاح الكوفيد-19 (كورونا) ؟	- نعم ☐<br>- كلا ☐

2- الخوف من الاصابة بفيروس كوفيد

يقوم هذا المقياس على قياس شدة خوفك اتجاه كوفيد-19(فيروس كورونا), رجاءا الرد على كل عنصر من خلال اختيار رقم من 1 الى 5 حسب ما يتوافق مع ما تشعر به أو تفكر فيه أو تتصرف به تجاه COVID-19

1- لا اوافق بشدة

2- لا أوافق إلى حد ما

3- لا أوافق ولا أرفض

4- أوافق إلى حد ما

5- أوافق بشدة

	لا أوافق بشدة	لا أوافق إلى حد ما	لا أوافق ولا أرفض	أوافق إلى حد ما	أوافق بشدة
1- أنا خائف من فيروس كورونا-١٩ لأقصى حدّ	1	2	3	4	5
2- التفكير بفيروس كورونا-١٩ يشعرني بعدم الارتياح	1	2	3	4	5
3- أشعر بتعرق في كفيّ عندما أفكر بفيروس كورونا-١٩	1	2	3	4	5
4- أخشى أن أفقد حياتي بسبب فيروس كورونا-١٩	1	2	3	4	5
5- ينتابني القلق أو التوتر عندما أشاهد أخبارًا أو قصصًا عن فيروس كورونا-١٩	1	2	3	4	5
6- لا يمكنني النوم بسبب قلقي من الإصابة بعدوى فيروس كورونا-١٩	1	2	3	4	5
7- تتسارع دقات قلبي عندما أفكر بالإصابة بعدوى فيروس كورونا-١٩	1	2	3	4	5

3- مقياس القلق من الموت

إقرأ من فضلك كل عبارة مما يلي بعناية،وقرر إلى أي حد تعد مشابهة لمشاعرك وسلوكك وآرائك خلال الفترة الحالية من انتشار فيروس كورونا في لبنان ، ثم بين مدى انطباقها او عدم انطباقها عليك بوجه عام وذلك من خلال اختيار احد الإجابات التالية :

1- لا

2- قليلا

3-متوسط

4- كثيرا

5- كثيرا جدا

	لا	قليلا	متوسط	كثيرا	كثيرا جدا
1- أخاف من الموت عندما يصيبني اي مرض	1	2	3	4	5
2- أخاف من النظر الى الموتى	1	2	3	4	5
3- أخاف من زيارة القبور	1	2	3	4	5
4- يرعبني احتمال ان تجرى لي عملية جراحية	1	2	3	4	5
5- أخاف من أن أصاب بنوبة قلبية	1	2	3	4	5
6- يقلقني أن يحرمني الموت من شخص عزيز عليّ	1	2	3	4	5
7- أخشى أمورا مجهولة بعد الموت	1	2	3	4	5
8- أخاف من رؤية جسد ميت	1	2	3	4	5
9- أخشى عذاب القبر	1	2	3	4	5
10- أخاف من ان اصاب بمرض خطير	1	2	3	4	5
11- ترعبني مشاهدة عملية دفن ميت	1	2	3	4	5
12- يرعبني السير بين المقابر	1	2	3	4	5
13- يشغلني التفكير فيما سيحدث بعد الموت	1	2	3	4	5
14- أخشى أن انام فلا أستيقظ أبدا	1	2	3	4	5
15- يرعبني الألم الذي يصاحب الموت	1	2	3	4	5
16- يزعجني مشاهدة جنازة	1	2	3	4	5
17- يخيفني منظر شخص يحتضر	1	2	3	4	5

18- يسبب لي الحديث عن الموت إزعاجاً	1	2	3	4	5
19- أخاف أن أصاب بالسرطان	1	2	3	4	5
20- أخاف من الموت	1	2	3	4	5

4- الضغط النفسي PHQ-9

كم عانيتَ(تِ) من المشاكل التالية خلال الأسبوعين الماضيين؟				
	أبدًا	بعض الأيام	اكثر من نصف الأيام	كل يوم تقريبًا
1- قلة الاهتمام أو الاستمتاع بممارسة الأشياء	0	1	2	3
2- الشعور بالحزن أو ضيق الصدر أو اليأس	0	1	2	3
3- الصعوبة في الركون إلى النوم أو النوم بانتظام أو النوم أكثر من العادة	0	1	2	3
4- الشعور بالتعب أو بقلة الحيوية	0	1	2	3
5- قلة شهية أو كثرة الأكل	0	1	2	3
6- الشعور بعدم الرضا عن النفس أو بالفشل أو الإحباط تجاه ذويك.	0	1	2	3
7- الصعوبة في التركيز على الأشياء،مثل قراءة الصحف أو مشاهدة التلفزيون	0	1	2	3
8- بطء في الحركة أو الكلام بدرجة ملحوظة للآخرين؟ أو على العكس كثرة التململ و التحرك إلى درجة فوق العادة	0	1	2	3
9- الشعو بتفضيل الموت عن الحياة، أو بإيذاء النفس بطريقةٍ ما	0	1	2	3

5- القلق GAD-7

خلال الأسبوعين الماضيين، كم مرة أقلقتكَ(كِ) المشاكل التالية؟				
	أبدًا	بعض الأيام	اكثر من نصف الأيام	كل يوم تقريبًا
1- الشعور بالغضب أو القلق أو الانفعال الشديد	0	1	2	3
2- عدم القدرة على إنهاء القلق أو التحكم فيه	0	1	2	3
3- القلق المفرط على أشياء مختلفة	0	1	2	3
4- الصعوبة في الاسترخاء	0	1	2	3
5- شدة الاضطراب لدرجة صعوبة البقاء في هدوء	0	1	2	3
6- السرعة في الانزعاج أو الانفعال	0	1	2	3
7- الشعور بالخوف كما لو أن فظيعًا قد يحدث	0	1	2	3
